# Early and late urodynamic assessment of the orthotopic N-shaped neobladder

**DOI:** 10.3892/ol.2013.1502

**Published:** 2013-07-30

**Authors:** ZONGLIANG ZHANG, HAIYAN QI, RONGXIANG ZHOU, XUNBO JIN

**Affiliations:** 1 Department of Urology, Qingdao Municipal Hospital, Qingdao, Shandong 266071, P.R. China; 2 School of Medicine, Shandong University, Jinan, Shandong 250012, P.R. China; 3 Minimally Invasive Urology Center, Provincial Hospital Affiliated to Shandong University, Shandong University, Jinan, Shandong 250021, P.R. China

**Keywords:** bladder neoplasms, neobladder, radical cystectomy, ileum, urodynamics, bladder substitution

## Abstract

The present study aimed to report the urodynamic data from 46 male patients who underwent an orthotopic N-shaped neobladder replacement following a radical cystectomy during a 12-month period and to provide urodynamic evidence for the improvement of this technique. A total of 46 consecutive male patients underwent a radical cystectomy and orthotopic N-shaped neobladder substitution due to muscle-invasive bladder cancer. Uroflowmetry, cystometry and urethral pressure profilometry were analyzed at 3–12 months following the surgery. The mean pressure of the neobladders was <15 cm H_2_O at volumes of <400 ml and 22.4 cm H_2_O at 100% capacity at 6 months. The mean pressure of the contractions was <40 cm H_2_O at 6 months. The mean filling pressure following the surgery at 3 months was higher compared with that at 6 months. No difference was observed at the other time-points. When comparing the 9- and 12-month urodynamic characteristics, no significant changes were observed in the cystometric capacity. The mean post-void residual (PVR) urine volume was 58 ml. A mean voiding peak of 16.2 ml/sec was obtained using the Valsalva maneuver. The daytime continence rate was 90% at 12 months while the night-time continence rate was 60%.

## Introduction

Orthotopic ileal neobladder construction following radical cystectomy is accepted as a novel method of urinary diversion. To date, numerous types of neobladders have been reported (1–5). The reservoir should be detubularized and compliant with a low end filling pressure (6,7). Daytime and night-time urinary incontinence, urethral or anastomotic strictures and a failure to empty the bladder substitute, thus requiring intermittent or permanent catheterization, may distract substantially from any perceived quality of life advantages of an orthotopic bladder reconstruction (8,9). The present study aimed to compare the early and late urodynamic assessment of the orthotopic N-shaped neobladder in patients who had undergone a radical cystectomy and ileal bladder substitution for muscle-invasive bladder cancer and to provide urodynamic evidence for the improvement of this technique.

## Materials and methods

### Patients and surgical technique

A total of 52 male patients with muscle-invasive bladder cancer who fulfilled the WHO bladder cancer criteria (2004 revision) were enrolled in the present study (10). The 52 patients with bladder cancer were confirmed using pathology examinations. The patients underwent a radical cystectomy and orthotopic N-shaped neobladder substitution and were scheduled for early (3 months) and late (12 months) urodynamic evaluations. Of the 52 patients, 6 succumbed to cancer, leaving 46 patients available for the early and late evaluations. The staging of the muscle-invasive bladder cancer was assessed using the TNM staging system for bladder cancer revised by the American Joint Committee On Cancer (AJCC) (11). All the patients were clinically staged as N0M0 pre-operatively by computerized tomography (Philips, Amsterdam, Holland) of the abdomen/pelvis, chest X-ray (Siemens, Munich, German) and nuclear bone scan (Siemens). The mean age of the patients was 60.9 years. Uroflowmetry, cystometry and urethral pressure profilometry were analyzed at 3–12 months following the surgery. Females were excluded from this analysis since the number of females with muscle-invasive bladder cancer is small, which may affect the functional outcome. All the patients provided their written informed consent prior to the initiation of the study.

**Figure 1:**
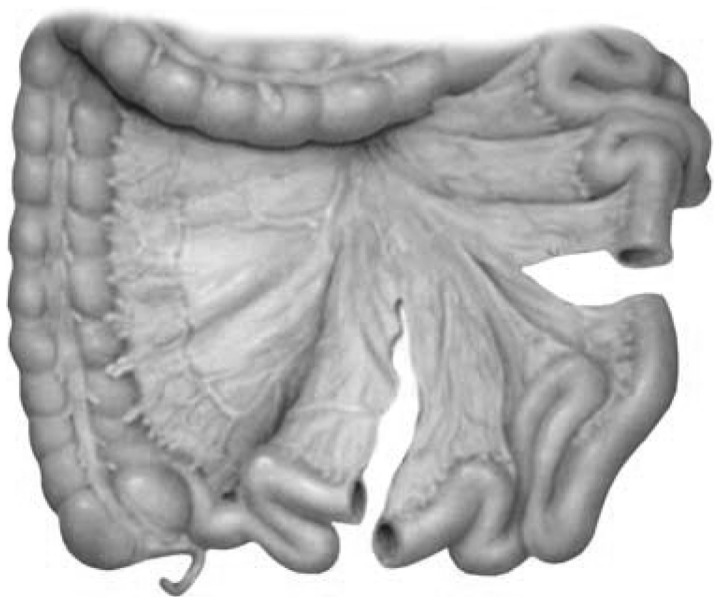
A 35-cm ileal segment was isolated 15–20 cm proximal to the ileocecalvalve.

**Figure 2:**
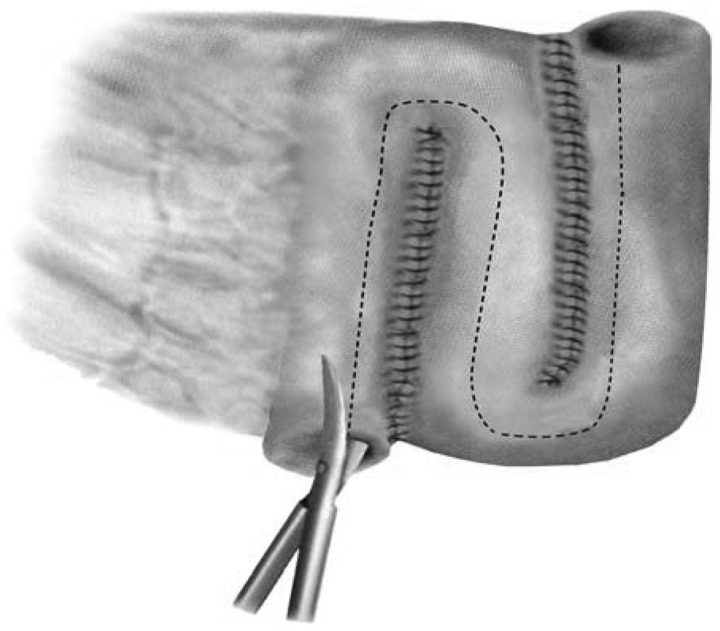
The distal loop (~35 cm in length) was lowered in a N shape. The ileal segment was then split open along the antimesenteric border.

**Figure 3:**
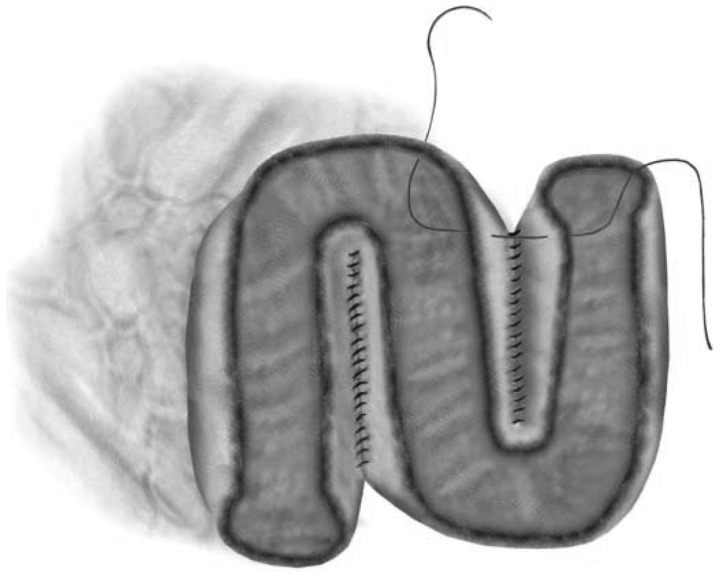
The proximal loop was folded in a reversed N shape and the inner opposite borders were then sutured side-to-side. This was tied to the opposite edge of the lower ileal segment to obtain an oval refashioned reservoir.

**Figure 4:**
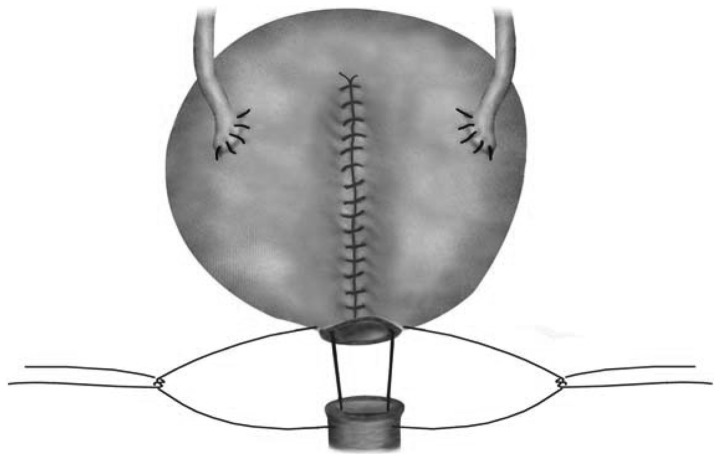
A ureteroileal anastomosis was performed bilaterally using the Nesbit technique in an open end-to-side fashion.

All the cystectomies and bladder substitutions were performed as previously described by Zhou *et al*(12). A 35-cm ileal segment was isolated 15–20 cm proximal to the ileocecal valve (Fig. 1). The length of the intestinal segment that was used for the reservoir was recorded at the surgery. The distal loop (~35 cm in length) was lowered in an N shape. The ileal segment was then split open along the antimesenteric border (Fig. 2). The proximal loop was folded in a reversed N shape and the inner opposite borders were then sutured side-to-side. This was tied to the opposite edge of the lower ileal segment to obtain an oval refashioned reservoir (Fig. 3). A ureteroileal anastomosis was performed bilaterally using the Nesbit technique in an open end-to-side fashion (Fig. 4).

### Urodynamic studies

The urodynamic analysis was performed using the BONITO UDS600 Urodynamic system (Laborie, Ontario, Canada). The urodynamic studies consisted of uroflowmetry, cystometry, urethral pressure profilometry and post-void residual (PVR) urine volume determination. Following the placement of a 10F transurethral dual channel catheter and a 10F rectal balloon catheter (Laborie, Mississauga, ON, Canada), the neobladder was filled at a rate of 50 ml/min with saline solution (Qingdao Huaren Pharmaceutical Co., Ltd., Qingdao, China) at room temperature. Uroflowmetry, cystometry and urethral pressure profilometry were analyzed at 3–12 months following the surgery. The parameters that were evaluated were the neobladder capacity, the pressure at the maximal capacity, urine leakage, the presence, amplitude and number of intrinsic contractions, the PVR urine volume and the urethral pressure profile. The maximum urine flow rate (Q_max_) was measured by uroflowmetry. The early (3, 6 and 9 months) and late (12 months) urodynamic results were compared.

### Follow-up

Each patient was followed up at 3, 6, 9 and 12 months post-operatively and then yearly in a prospective protocol that included clinical, metabolic and radiological assessment from the standpoint of the oncological follow-up and lower urinary tract function. In the case of elevated serum creatinine levels or hydronephrosis, a renal scan using technetium-99m (^99m^Tc)-diethylenetriamine-pentaacetic-acid was performed.

To assess the lower urinary tract function in detail, voiding and continence diaries (frequency/volume chart for 2–3 days) and a standardized questionnaire were completed at 3, 6, 9 and 12 months following the surgery and annually thereafter. The questionnaire assessed the presence and degree of daytime and night-time urinary incontinence; described as mucus only, few drops, a spoonful, approximately half a glass, a glass full and almost all. The questionnaire also analyzed the frequency of daytime and night-time urinary incontinence episodes and the number of absorbent pads used day and night. Full continence was defined as daytime and night-time dryness without the requirement for a pad or condom catheter.

### Statistical analysis

The statistical analysis was performed using SPSS 13.0 software (SPSS, Inc., Chicago, IL, USA). The correlation analysis was performed using the Pearson correlation test. The data are expressed as the mean ± standard deviation. P<0.05 was considered to indicate a statistically significant difference between the early and late urodynamic assessments.

## Results

The mean age of the patients was 60.9±7.6 years (range, 49–72 years). The mean pressure of the neobladders was <15 cm H_2_O at volumes of <400 ml and 22.4 cm H_2_O at 100% capacity at 6 months. The mean pressure of the contractions was <40 cm H_2_O at 6 months. The mean filling pressure following the surgery was relatively stable. The results demonstrated that the mean filling pressure levels were significantly higher at 3 months than at 6 months following the surgery. The differences between the levels at 6, 9 and 12 months showed no statistical significance. When comparing the urodynamic characteristics at 9 months with those at 12 months, no significant changes were observed in the cystometric capacity. The mean PVR volume was 58 ml. A mean voiding peak of 16.2 ml/sec was obtained using the Valsalva maneuver. The daytime and night-time continence rates were 90 and 60%, respectively, at 12 months. The questionnaires concerning the quality of life revealed no difference between the patient group and healthy group. The urodynamic evaluation is shown in Table I.

## Discussion

Neobladder substitution has currently become the standard for urinary diversion due to good long-term functional results (13–15). Various segments of bowel have been used extensively for bladder augmentation or replacement, including detubularized ileum, detubularized sigmoid colon and the right colon with or without a patch of ileum. When measuring the quality of life of patients with orthotopic bladder substitutions, functional voiding is a significant factor (8,9). The present study aimed to compare the early and late urodynamic assessments of the orthotopic N-shaped neobladder in patients who had undergone a radical cystectomy and ileal bladder substitution for muscle-invasive bladder cancer and to provide urodynamic evidence for the improvement of this technique.

The success of orthotopic bladder substitutions to store urine, and thus provide urinary continence, relies on a sufficient functional reservoir capacity, detubularization of the intestinal segment to reduce intravesical pressure and the preservation of the external urethral sphincter tonus (6,7). The reservoir should be detubularized and compliant with a low end filling pressure (6,7). The detubularization and overfolding of the ileal segment creates the maximum radius that concomitantly results in a four-fold increase in the volume. The mathematical model offered by Hinman (16) and animal studies conducted by Shaaban *et al*(17) have shown that the mainstay of creating a low-pressure, high-volume reservoir is by the detubularization and reconfiguration of the intestinal segments. In the present study, the mean maximum cystometric pouch capacity was 526 ml and the pressure levels during the filling phase of the orthotopic bladder were relatively low. Only a 30-cm ileal segment was used for the reconstruction of the neobladder. Compared with other procedures, the length of the intestinal segment that was used for the reservoir was the shortest. The mean pressure of contractions was <40 cm H_2_O. In a native bladder, the pressures are usually <5–10 cm H_2_O during the filling cystometry. However, in a neobladder of intestinal origin, it is accepted that pressures of up to 40 cm H_2_O may be safely tolerated in terms of preserving the upper urinary tract functions. Therefore, with its low pressure, the N-shaped neobladder appears to present no risk to the upper urinary tract. The present study identified that the mean filling pressure following the surgery was relatively stable, with the exception of at 3 and 6 months. The differences between the 6-, 9- and 12-month time-points showed no statistical significance. In the present study, the maximal capacity was stable at 6 and 12 months and the PVR urine volume was <100 ml in the majority of the cases, thus showing that the reservoir capacity remained stable at 6 months. The high compliance of the ileal neobladder is the main factor in achieving near-normal voiding patterns and preserving the upper urinary tract. Bachor *et al*(18) stated that high compliance levels were associated with the preservation of the upper urinary tract with a pouch pressure of 25 cm H_2_O. In the present study, the group mean Q_max_ value and PVR urine volume were 16.2 ml/sec and 58 ml, respectively. Overdistension with a loss of wall tension should be avoided, as it may produce incomplete emptying. Porru *et al*(19) observed that only patients with a capacity of >700 ml had a significant PVR urine volume (>100 ml). In the present study, the maximal capacity was stable at 6 months and the PVR urine volume was <100 ml in the majority of the cases.

In the present study, orthotopic neobladder replacement following radical cystectomy was well accepted by the patients and provided a good health-related quality of life as a result of a near-normal daytime and night-time continence status. Continence following orthotopic urinary diversion is dependent on an intact urethral sphincter function and an intact pelvic floor, which are able to maintain a resistance pressure across the urethral continence zone that exceeds the pressure generated within the diversion (9,20,21). Additional factors that may affect continence include the urethral length and sensitivity, the patient age and mental status, an intact pelvic nerve supply to the rhabdosphincter, the completeness of voiding and the presence or absence of bacteriuria (22). Early (median follow-up, 9 months) and long-term (median follow-up, 62 months) daytime continence rates have been reported as 80 and 90% (23), respectively. In the present study, the daytime continence rate was 90%, which is in parallel with other studies.

The night-time incontinence of a neobladder occurs due to the absence of a neurological feedback and sphincter detrusor reflex, the relaxation of the pelvis and a decreased sphincter tonus at night (9). The night-time continence rate in the present study was 60% and therefore below the rates that have been reported in other studies (18,21). Nocturnal continence has a statistically significant positive correlation with the maximal urethral closure pressure and a negative correlation with the maximal contraction amplitude and the pressures at mid- and maximal capacity (24). Age is also a contributing factor in the establishment of continence. Careful patient selection is as significant as the surgical technique that is applied during the bladder substitution procedure in order to achieve a good continence status. To reduce enuresis, patients may be instructed to limit fluid intake following an evening meal, to void prior to going to sleep and to set on an alarm clock to awaken and void once or twice during the night. This education is aimed at reactivating the perception of the desire to void during sleep to obtain an improved control of the neobladder (23).

With regard to the quality of life of the patient, no evidence has been identified to support an advantage of one type of orthotopic reconstruction over another (25). In the present study, the majority of the aspects of life quality, including the urinary symptoms and continence rate, were evaluated using the European Organisation for Research and Treatment of Cancer (EORTC) QLQ-C30 and BLM30 questionnaires and the results were judged as excellent.

In the present study, the orthotopic N-shaped neobladder maintained stable urodynamic parameters and function and was associated with a good quality of life. The urodynamic parameters of the neobladder were similar to those of a normal urinary bladder. The construction of an orthotopic continent globular ileal bladder required a shorter ileal segment compared with those of other similar procedures. According to the present data, the orthotopic N-shaped neobladder is a valid treatment option for treating muscle-invasive bladder cancer.

## Figures and Tables

**Table I:** Urodynamic evaluation of 46 male patients.

	Follow-up (months)			
	
Urodynamic index	3	6	9	12
Maximum bladder capacity (ml)	386.0±46.6	403.5±49.7	414.9±45.0	444.9±97.0
PVR urine volume (ml)	41.3±10.6	38.5±4.6	36.2±8.9	32.4±7.9
Flow (ml/sec)	14.0±2.0	16.2±2.2	17.6±4.8	19.9±2.6
Pressure at maximum capacity (cm H_2_O)	21.4±3.6	18.2±4.0	17.7±3.1	16.5±3.7
Pressure at Q_max_ (cm H_2_O)	46.2±4.7	48.9±4.7	52.7±4.8	53.2±5.5

Data are presented as the mean ± standard deviation unless stated. PVR, post-void residual; Q_max_, maximum urine flow rate.
